# Costs and benefits of predator-induced defence in a toxic diatom

**DOI:** 10.1098/rspb.2021.2735

**Published:** 2022-04-13

**Authors:** Anna J. Olesen, Fredrik Ryderheim, Bernd Krock, Nina Lundholm, Thomas Kiørboe

**Affiliations:** ^1^ Natural History Museum of Denmark, University of Copenhagen, Øster Farimagsgade 5, 1353 Copenhagen K, Denmark; ^2^ Centre for Ocean Life, DTU Aqua, Technical University of Denmark, Kemitorvet, Building 202, 2800 Kgs. Lyngby, Denmark; ^3^ Alfred Wegener Institut-Helmholtz Zentrum für Polar- und Meeresforschung, Chemische Ökologie, Am Handelshafen 12, 27570 Bremerhaven, Germany

**Keywords:** diatom defence, copepods, marine chemical interactions, defensive benefit, trade-offs

## Abstract

Phytoplankton employ a variety of defence mechanisms against predation, including production of toxins. Domoic acid (DA) production by the diatom *Pseudo-nitzschia* spp. is induced by the presence of predators and is considered to provide defence benefits, but the evidence is circumstantial. We exposed eight different strains of *P. seriata* to chemical cues from copepods and examined the costs and the benefits of toxin production. The magnitude of the induced toxin response was highly variable among strains, while the costs in terms of growth reduction per DA cell quota were similar and the trade-off thus consistent. We found two components of the defence in induced cells: (i) a ‘private good’ in terms of elevated rejection of captured cells and (ii) a ‘public good’ facilitated by a reduction in copepod feeding activity. Induced cells were more frequently rejected by copepods and rejections were directly correlated with DA cell quota and independent of access to other food items. By contrast, the public-good effect was diminished by the presence of alternative prey suggesting that it does not play a major role in bloom formation and that its evolution is closely associated with the grazing-deterrent private good.

## 1. Introduction

The herbivory pressure in pelagic ecosystems is intense, with up to three times higher fraction of the production being consumed compared to terrestrial systems [1]. In response, phytoplankton have evolved numerous defence mechanisms, varying from morphological and behavioural to physiological and including the production of various chemical substances [2]. Through the evolution of costly defence mechanisms, grazing may be an important driver of diversity in phytoplankton communities by allowing coexistence of defence and competition specialists [3–5]. Coexistence requires that defence benefits come with a cost, e.g. in the form of reduced growth [6], but both benefits and costs of defences in phytoplankton have rarely been convincingly demonstrated [2].

Toxic algal blooms are a global phenomenon. Often, the toxins accumulate in the food web and may negatively affect several trophic levels [7]. Diatoms of the cosmopolitan genus *Pseudo-nitzschia* can produce the neurotoxin domoic acid (DA) and form dense toxic blooms with severe consequences to marine organisms and ecosystems, like mass death incidents of seabirds and marine mammals [7,8]. However, the potential benefits and costs of DA production have not been established. There is conflicting evidence on whether DA production is costly; some studies have suggested that cells with increased toxin content have lower growth rates [9,10], but other experiments have been unable to reproduce these results [11–13]. Similarly, there is only circumstantial evidence that DA acts as a predator-deterrent. Some studies have demonstrated reduced predator activity in zooplankton exposed to toxic *Pseudo-nitzschia* [11,14]. Whether this was due to predators becoming intoxicated after consuming some cells or being exposed to dissolved DA [15], or whether the grazers actively avoid eating toxic cells is in all cases unclear (a toxic versus a deterrent effect). However, toxic effects are ‘public goods’ [16] that also benefit non-toxic competitors, and ‘cheaters’ that do not pay the probable cost of producing the toxin may outcompete the producers. The evolution of such public goods, unless associated with private benefits, is not evolutionary supported. By contrast, the evolution of toxin production as a ‘private good’, where only the cell producing the toxin benefits from it, is easy to envisage [16,17].

Further evidence of trade-offs and a defensive value of DA is that its production is induced by chemical cues from copepods, copepodamides [9,18]. Defences are expected to be inducible only if threats are varying, and the defence is costly [19]. If defences did not have associated costs, all organisms would evolve towards an equal state of defence [20].

Here, we explore the defensive value and trade-offs of DA production in *P. seriata* exposed to different concentrations of toxin-inducing copepod cues. We include eight different strains to account for possible intraspecific variation. We use direct video observations of individual copepod–prey interactions to quantify the defensive benefits of DA in terms of captured cells being consumed or rejected, and we quantify the costs of DA production from changes in cell division rate. We demonstrate significant benefits and costs of DA production and thus a clear trade-off. We further demonstrate threefold differences in growth rate among strains, but that the trade-off of DA production is the same. We finally show that DA production provides both private and public goods, thus suggesting a potential mechanism for the evolution of apparently public goods.

## 2. Material and methods

### (a) Culturing and species identification

To establish cultures of *Pseudo-nitzschia* for the experiments, live phytoplankton samples were collected in Øresund, Denmark (55°45′40″ N 12°36′0″ E), in April 2020, by hauling a 20 µm plankton net through the upper (0–5 m) water column. Single cells or chains were micro-pipetted and placed individually into 96-well plates with L1 medium [21] using a light microscope (CKX53, Olympus, Tokyo Japan). The cultures were kept at 4°C at a 16 : 8 h light : dark cycle and 120 µmol photons m^−2^ s^−1^ using cool white light bulbs. Identification was performed by transmission electron microscopy (JEOL, JEM 1010, Tokyo, Japan) following Hasle & Lundholm [22]. All strains included in this study were identified as *P. seriata* (electronic supplementary material, tables S1 and S2).

### (b) Induction and cost of toxin production

To quantify the costs of toxin production, we performed dose–response experiments with eight strains of *P. seriata*, where we measured the growth rate as a function of copepodamide concentration and consequent cellular contents of DA. Cells of different strains were suspended in L1 medium and added to 200 ml cell-culture flasks. The flasks were exposed to copepodamides extracted from freeze-dried *Calanus* spp. [23] by coating the inside of the wall with a mixture of copepodamides dissolved in methanol. The methanol was evaporated using N_2_ gas. Controls underwent the same treatment but received methanol without copepodamides. Six strains of *P. seriata* were exposed to 0, 10 or 50 pM of copepodamides, while two strains were exposed to 0 or 50 pM. The flasks were incubated for 72 h at 4°C or 8°C (only strain SKC620) and *ca* 100 µmol photons m^−2^ s^−1^ on a 14 : 10 light : dark cycle. Due to slow release and quick degradation, the copepodamides coated on the inside of the bottles typically yield an actual concentration of 1% of the added amount after 12 h, and much less after 72 h [18]. After 72 h incubation, the cells were harvested for enumeration of cell densities and quantification of cellular DA content. Cell densities were determined by fixing 3 ml of sample in acidic Lugol's solution (final concentration 2–3%) and counting a minimum of 400 cells in a Sedgewick-Rafter chamber using an inverted microscope. Cell growth rates were calculated using temporal variations in cell densities assuming exponential growth. Average cell size was determined by measuring 25–30 random cells and cell volume calculated based on Lundholm *et al*. [24]. For five of the strains, we also measured dissolved DA. Additional cells from the two strains exposed to only 0 or 50 pM were harvested for use in copepod foraging experiments.

### (c) Copepod foraging experiments

We directly observed the feeding response of copepods to the cells by video recording individual copepod–cell interactions. The feeding experiments were done in a constant temperature room (8°C) in darkness. Adult females of the feeding-current feeding copepod *Temora longicornis* were glued by their dorsal surface to a human hair [25,26] and starved overnight in darkness. The other end of the hair was attached to a micromanipulator and the copepod was submerged in a 10 × 10 × 10 cm^3^ aquarium with filtered seawater (salinity [27]). A different copepod was used for each replicate in each treatment and experiment (three copepods per treatment). *Pseudo-nitzschia* cells were added to the aquarium and the copepod response was immediately recorded using a high-speed camera (Phantom V210, Vision Research, New Jersey, USA). The camera was equipped with lenses to yield a field of view of about 1.3 × 1.0 mm^2^. A magnetic stirrer gently mixed the water and kept the cells in suspension.

We used two of the strains in the feeding experiments (SKC620 and AI420). The copepods were fed either non-induced control cells or cells that had been exposed to 50 pM of copepodamides for 72 h, as described above, to yield different toxin cell quotas. We conducted three types of experiments: (i) in feeding experiments with strain SKC620, we recorded four 5 min (0–5 min, 20–25 min, 35–40 min, 55–60 min after adding cells) sequences at 50 frames per second (fps) at a cell density of 200 cells ml^−1^. (ii) In a similar feeding experiment with strain AI420, we recorded six 3 min (0–3 min, 10–13 min, 20–23 min, 30–33, 40–43, 50–53) sequences at 100 fps with the same cell density as above. (iii) In a third experiment, we mixed toxic *P. seriata* (strain AI420) with the non-toxic diatom *Ditylum brightwellii*, 100 cells ml^−1^ of each species and recorded sequences (at 100 fps) as above. The two species were easy to distinguish in the videos. The video recordings were analysed to quantify copepod beating activity and frequency, prey capture and the fraction of captured cells that were rejected by the copepods. Additional videos of five strains were recorded using the same set-up but with 1000 fps to examine potential differences in prey-handling time.

### (d) Toxin analysis

In all experiments, 45 or 60 ml of each replicate was harvested and divided into 15 ml centrifuge tubes and centrifuged at 760*g* for 15 min. The supernatants of five strains (B2N, E9N, D2N, G3N and H4N) were kept in 15 ml falcon tubes and stored frozen at –20°C for analysis of dissolved DA. For intracellular toxins, cell pellets were pooled in 2 ml cryotubes and centrifuged again at 1507*g* for 15 min. The pellets were stored frozen at –20°C. Toxins were extracted and analysed using liquid chromatography coupled with tandem mass spectrometry [27].

All raw data are available in Olesen *et al*. [28].

### (e) Statistical analyses

The effect of the copepodamide treatment on the fraction of time spent beating was analysed by a mixed-effects logistic regression using the *lme4* R-package [29]. ‘Treatment’ and ‘sequence’ were used as fixed effects and ‘replicate’ as the random effect. Pairwise comparisons between the different treatments (control–induced, or control–induced–mix) were done using the Satterthwaite degrees-of-freedom method. The relationship between growth rate or prey-handling time and cellular DA content was analysed using a linear mixed-effects model (again using *lme4*) with ‘strain’ as the random effect. All statistical models were validated by visual inspection of residual plots.

## 3. Results

### (a) Induction and cost of toxin production

**Figure 1.**
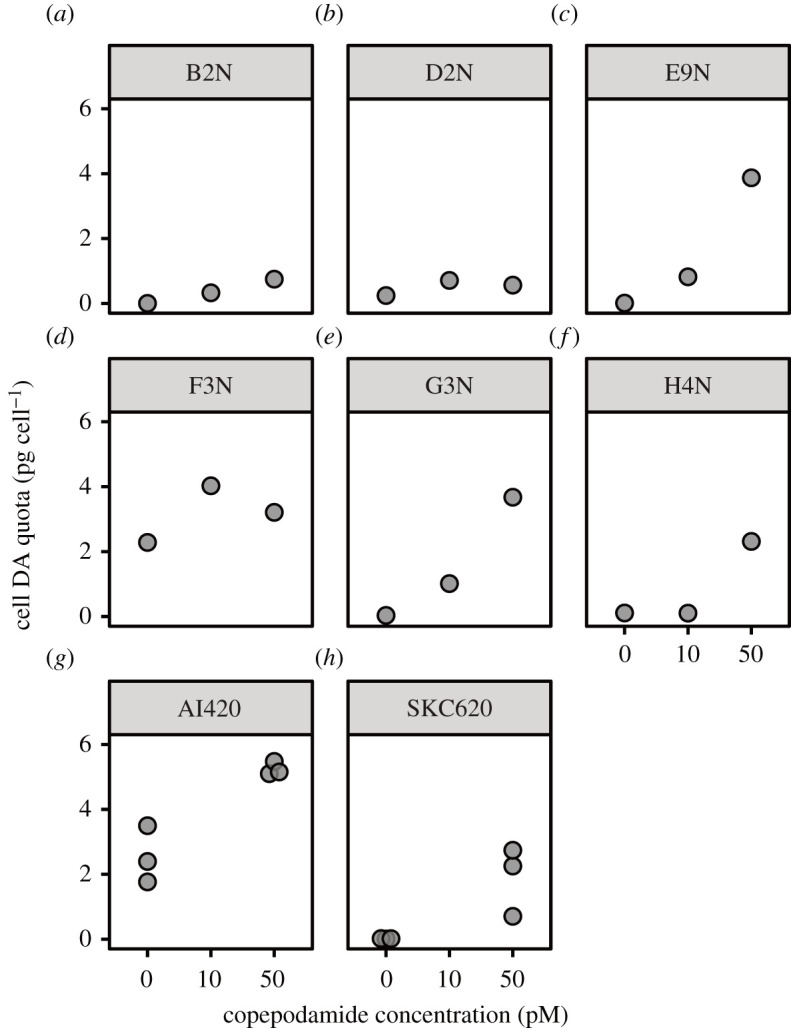
Dose–response experiments. Cellular DA content (pg cell^−1^) as a function of the nominal copepodamide concentration for the eight different strains of *P. seriata*. For each concentration in (*a*–*f*), *n* = 1, and *n* = 3 for each concentration in (*g*–*h*).

Exposure to copepodamides increased the cellular toxin content in *P. seriata*, but there was a large variation among strains in terms of the magnitude of the response (figure 1). The relative increase of cellular DA content in the induced treatments compared to the non-induced control was between 40% and 35 000% (figure 1; electronic supplementary material, table S1). In the five tested strains (B2N, D2N, E9N, G3N and H4N), cellular and dissolved DA were not significantly correlated (linear regression, *F*_5,9_ = 0.6, *p* = 0.72).

**Figure 2.**
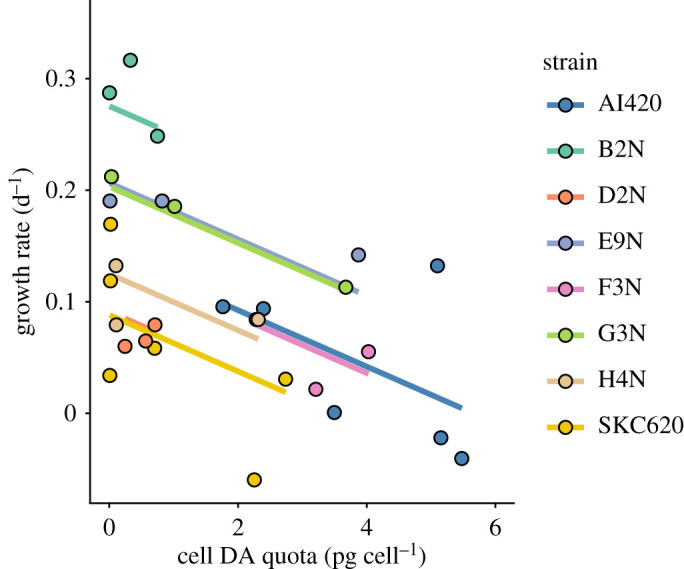
Growth–defence trade-off. Growth rate as a function of cellular DA content for all strains used in this study. A linear mixed-effects model was fit to the data with one slope and random intercepts. The estimated slope (with 95% confidence intervals) is –0.025 d^−1^ (pg DA cell^−1^)^−1^ [–0.032, –0.018] (*F*_1,25.3_ = 13.2, *p* < 0.001).

Growth rates generally decreased significantly with increasing toxin content (figure 2; electronic supplementary material, table S1). While the intercepts differed significantly among strains, suggesting a threefold variation in growth rate, the slopes did not differ significantly from one another. Thus, the cost of DA is the same for all the strains (figure 2).

### (b) Copepod foraging response

*Temora longicornis* copepods use their feeding appendages to create a feeding current from which prey particles are individually perceived and captured. The copepods react to *Pseudo-nitzschia* cells that are close to or touching the copepod feeding appendages. Depending on cell orientation, the copepod briefly adjusts the captured particle and brings the cell or chain to the mouth (electronic supplementary material, videos S1 and S2). Prey handling by the feeding appendages concludes with the cell either being ingested or rejected (electronic supplementary material, videos S1 and S2). The time spent handling the ingested particles increased significantly with chain length (electronic supplementary material, figure S1) but was independent of cellular DA content.

**Figure 3.**
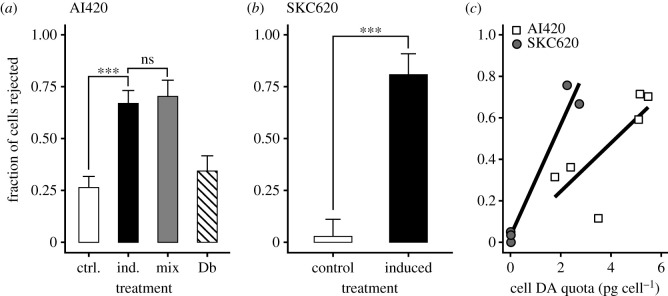
Defence efficiency. The fraction of cells rejected in strain (*a*) AI420 and (*b*) SKC620. Mix: the fraction of induced *P. seriata* cells rejected in the mixed experiment. Db: fraction of *D. brightwellii* cells rejected in the mixed experiment. (*c*) Relationship between the fraction of rejected cells and the cellular DA content. Values in (*a*) and (*b*) are the fractions from all captures across several copepods recorded and error bars show 95% Wilson score interval (*n* = 48–287). In (*c*), they are the fractions for each copepod and include the control and induced treatments. One data point was removed in (*c*) due to the copepod only capturing two cells (both were rejected). Asterisks indicate significant difference between treatments, **p < 0.01, ***p < 0.001. ns: not significant. Odds ratios (with 95% confidence intervals) when comparing ‘control’ and ‘induced’ treatments are (AI420) 5.71 [3.86, 8.54] and (SKC620) 83.99 [21.76, 563.50].

The two strains used in the foraging experiments both responded to the copepodamides by increasing their cellular DA content (figure 1*g*,*h*; two-way ANOVA, *F*_3,8_ = 29.0, *p* = 0.01), and a significantly larger fraction of the induced than the control cells caught by the copepods was rejected (figure 3*a*–*c*). Overall, the fraction of rejected cells increased with the cellular DA content (figure 3*c*). The addition of the alternative prey (*D. brightwellii*) in the mixed experiment with strain AI420 did not affect the fraction of *P. seriata* cells rejected (figure 3*a*). The *D. brightwellii* cells were rejected at a rate similar to that of the non-induced *P. seriata* (figure 3*a*).

**Figure 4.**
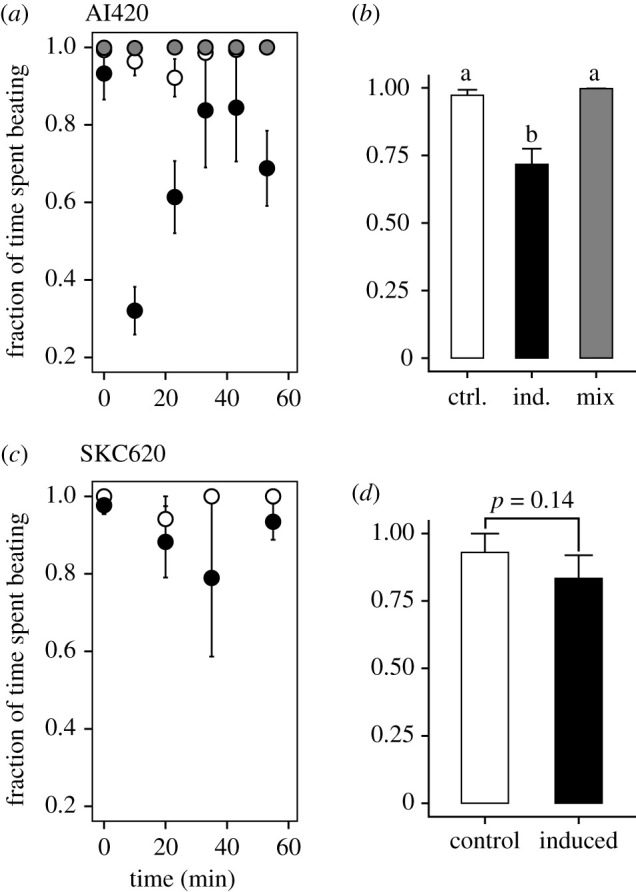
Copepod beating frequency. Fraction of time the copepods spent beating starting as the cells are added (*a*,*c*) and averaged for each treatment (*b*,*d*) in the foraging experiments with strains AI420 (top row) and SKC620 (bottom row). Lower case letters refer to significant differences between treatments according to pairwise comparisons (*p* < 0.05). Colours referring to treatments in the bar charts (*b*,*d*) apply also in (*a*) and (*c*). Error bars in all panels show standard error (*n* = 3, except in ‘Mix’ where *n* = 2).

The copepods were initially beating their appendages 100% of the time irrespectively of treatment (figure 4*a*,*c*). However, within minutes, exposure to the induced *P. seriata* cells (AI429) significantly reduced the fraction of time that the copepods spent creating a feeding current (figure 4*a*,*b*). Beating time was, however, not different from the controls when the induced treatment was fed to the copepod in conjunction with *D. brightwellii* (figure 4*a*,*b*). When exposed to induced *P. seriata* SKC620, a similar trend was observed (figure 4*c*,*d*), but the difference was not statistically significant.

## 4. Discussion

Our results show for the first time a convincing enhancement of survival in diatoms with induced toxin production when preyed upon by copepods. The survival strategy comes with the costs of a significant decrease in growth rate.

### (a) The defensive benefit of domoic acid

The copepods individually examined captured prey before deciding to ingest or reject it. This mechanism is similar to that of copepods feeding on toxic dinoflagellates [30], but it is still unclear how the copepods sense the toxicity of phytoplankton cells without, presumably, harming them.

The increased odds of cell rejection in the induced treatment is a ‘private good’ that only cells that produce the toxin benefit from [16]. This is similar to the effect of toxins produced by *Alexandrium* spp. [26,30]. However, in addition, we find that the feeding activity of the copepods was reduced when offered only induced *P. seriata*. This would be a ‘public good’ [16] because the cells that are not producing the costly toxins also benefit. This effect may be due to a harmful effect of the dissolved DA, which has been found to reduce feeding in krill [15], or it may be due to some of the toxic cells being eaten and intoxicating the copepod. The latter may explain why some time elapses before the effect is noticeable [11,25]. Public goods are in most cases not considered evolutionary stable strategies [16,31]. However, if associated with a private good, as suggested here, one can envisage a mechanism for their evolution. The public-good effect is diminished by the presence of alternative prey and would thus only become ‘public’ in the later stages of a bloom when toxic cells presumably dominate, and thus not promote bloom formation [32].

The private-good defence is measured as the fraction of captured cells that are rejected by the copepod. Up to 75% of induced cells were rejected after capture; hence, toxin production may increase the fitness of the cell provided that the costs are less than the benefits. While the absolute cost of toxin production is similar among the examined strains, the relative cost is much larger for slowly growing strains. Thus, in slowly growing SKC620, the relative reduction in growth rate of induced cells, approximately 75%, is of the same magnitude as the 75% reduction in predation risk, while in fast-growing strains, the benefits outweigh the costs. This suggests that there are strong trade-offs related to variation in growth rate. Otherwise, fast-growing strains with a very favourable cost–benefit trade-off for toxin production would rapidly outcompete slowly growing cells with a very limited fitness benefit of toxin production.

The presence of an alternative prey may influence copepod prey selection and contribute to the relative increase of toxic cells [33,34]. However, capture rate (normalized by cell density) of *P. seriata*, and subsequently the potential grazing mortality, doubled with the addition of *D. brightwellii* compared to when only toxic cells were present as prey because the copepods did not reduce their beating activity. The addition of the *D. brightwellii* cells did not change the fraction of induced *P. seriata* cells that were rejected. Thus, responding to predator presence by increasing toxin production is still an adequate response by *P. seriata*, and presumably also other toxic phytoplankton, in mixed prey suspensions. This is similar to the effect of prey concentration on the rejection frequency of three species of centric diatoms that thicken their shells in response to copepod cues [35]. A higher prey concentration increased the odds of rejection in both defended and undefended cells, but the relative increased odds of rejection in defended cells remained similar.

The inability of previous studies to show evidence of a private-good defence in *Pseudo-nitzschia* spp. may stem from the use of wild copepods, or as an artefact of the experimental design (‘black-box’ incubations). The present study is the first to use direct observations of copepod–*P. seriata* interactions [11,12,36]. Predators that are frequently exposed to the presence of toxic cells may be more tolerant to the toxins [37,38], and the build-up of resistance is quick, i.e. over a few generations [39]. Thus, the collection of wild copepods, e.g. during or towards the end of a bloom, may influence the results. The copepods used in this study were originally isolated from the same location as the *P. seriata* strains, but have since been kept in culture for many generations leading, presumably, to a loss of tolerance to DA. However, our results may still be ecologically relevant. *T. longicornis* generally produce approximately six generations per year [40,41], in contrast with the longer generation times of *Calanus* spp. used in several earlier studies [42,43]. Jiang *et al*. [44] found that *Acartia tonsa* resistance to the toxic dinoflagellate *Cochlodinium polykrikoides* was relaxed after just two generations of non-exposure. Thus, the loss of tolerance may be fast once toxic cell abundance declines [45,46], and the defence will be efficient in time for the next blooming event.

### (b) The cost of toxin production

Defence trade-offs in phytoplankton are notably difficult to establish [2]. However, the fact that many phytoplankton defences are inducible—they respond to predator cues by upregulating their defence—suggests that costs should be present [19,47]. We find here a clear trade-off between predator-induced toxin production and prey growth rate, and the costs were similar among the tested strains.

Several previous experiments have been unable to measure the costs of toxin production in *Pseudo-nitzschia* (e.g. [10,27]). Lundholm *et al*. [9] speculated that this could be due to low levels of DA that are insufficient to imply measurable differences in growth rates. However, the trade-off becomes clear in our experiments with the higher levels of DA induced by copepodamide exposure (figure 2). Selander *et al*. [18] measured the *in situ* concentration of copepodamides over the course of a year and found values ranging between 40 and 2000 fM. Thus, the concentrations used in our experiments are within the natural range, and the reduction in growth rate observed due to increased toxin production is ecologically relevant.

The direct metabolic cost of DA synthesis (see electronic supplementary material, information) is low and just 1–2% of the growth reduction can be accounted for by energy allocated directly to DA production (electronic supplementary material, figure S2). This suggests that other metabolic processes linked to toxin production are important. Phytoplankton exposed to predators up- or downregulate thousands of genes, e.g. genes related to signal transduction pathways, stress responses, and lipid and nitrogen metabolism [48,49]. Such associated responses may be energetically costly and may account for the growth reduction.

### (c) Toxic blooms and ecosystem implications

The formation of toxic algal blooms have implications for marine ecosystems [7,8]. The ultimate cause of such blooms is still debated [50] but is frequently attributed to eutrophication. The ‘private-good’ mechanism demonstrated here improves our comprehension of such toxic blooms and the factors that allow them to form. A mechanistic understanding of this is key to predicting their occurrence and how they may be affected by a changing climate.

## Data Availability

Data are available from: https://doi.org/10.5281/zenodo.6346438. The data are provided in the electronic supplementary material [51].
